# Pregnancy‐adapted uterine artery endothelial cell Ca2+ signaling and its relationship with membrane potential

**DOI:** 10.14814/phy2.13452

**Published:** 2017-11-09

**Authors:** Roxanne E. Alvarez, Derek S. Boeldt, Bikash R. Pattnaik, Hannah L. Friedman, Ian M. Bird

**Affiliations:** ^1^ Perinatal Research Laboratories Department of Obstetrics and Gynecology University of Wisconsin Madison Wisconsin; ^2^ Department of Pediatrics University of Wisconsin Madison Wisconsin; ^3^ Department of Ophthalmology and Vision Sciences University of Wisconsin Madison Wisconsin

**Keywords:** Adaptation, Ca2+ signaling, endothelial, membrane potential, pregnancy

## Abstract

Pregnancy‐derived uterine artery endothelial cells (P‐UAEC) express P2Y2 receptors and at high cell density show sustained and synchronous [Ca2+]i burst responses in response to ATP. Bursts in turn require coupling of transient receptor potential canonical type3 channel (TRPC3) and inositol 1,4,5‐triphosphate receptor type 2 (IP3R2), which is upregulated in P‐UAEC in a manner dependent on connexin 43 (Cx43) gap junctions. While there is no known direct interaction of TRPC3 with Cx43, early descriptions of TRPC3 function showed it may also be influenced by altered membrane potential (*V*_m_). Herein, we ask if enhanced TRPC3 Ca2+ bursting due to enhanced Cx43 coupling may be coupled via dynamic alterations in *V*_m_ in P‐UAEC, as reported in some (HUVEC) but not all endothelial cells. Following basic electrical characterization of UAEC, we employed a high sensitivity cell imaging system to simultaneously monitor cell *V*_m_ and [Ca2+]i in real time in continuous monolayers of UAEC. Our findings show that while acute and sustained phase [Ca2+]i bursting occur dose‐dependently in response to ATP,* V*_m_ is not coregulated with any periodicity related to [Ca2+]i bursting. Only a small but significant progressive change in *V*_m_ is seen, and this is more closely related to overall mobilization of Ca2+. Surprisingly, this is also most apparent in NP‐UAEC > P‐UAEC. In contrast [Ca2+]i bursting is more synchronous in P‐UAEC and even achieves [Ca2+]i waves passing through the P‐UAEC monolayer. The relevance of these findings to mechanisms of pregnancy adaptation and its failure in hypertensive pregnancy are discussed.

## Introduction

Maternal vascular adaptations are a necessary underlying component of a healthy pregnancy and include enhanced vasodilation. Nonetheless, in preeclamptic pregnancies, vascular adaptation is insufficient and is associated with impaired endothelial vasodilatory function and hypertension (Hladunewich et al. 2007). Using cells isolated from pregnant (P) and nonpregnant (NP) ewes, we have previously established uterine artery endothelial primary cell culture model (UAEC) in which early passage P‐UAEC are shown to retain pregnancy‐specific cell signaling adaptations which support enhanced vasodilator production, including nitric oxide (NO). In Gifford et al. (2006a), showed pregnancy‐adapted Ca2+ signaling and NO production were the result of greater capacitative Ca2+ entry in P‐UAEC. These enhancements were not due to expressional differences in key regulatory proteins such as purinergic (P2X or P2Y) receptors, G‐alpha proteins, inositol 1,4,5‐trisphosphate receptor (IP3R), or transient receptor potential (TRP) type C3 or C6 proteins. Rather, a greater ATP‐driven IP3R2‐TRPC3 *interaction* occurred in agonist‐stimulated P‐UAEC that was otherwise lacking in NP‐UAEC (Gifford et al. 2006b). In 2009, our move to video imaging allowed the simultaneous study of [Ca2+]i signaling and NO production in P‐UAEC at high cell density and the phenomena of [Ca2+]i bursting became increasingly evident at higher doses of ATP (Yi et al. 2010). Since that time, Yi et al. (2010) further showed the pregnancy‐adapted Ca2+ signaling in P‐UAEC could be observed over 30 min. In response to ATP stimulation, an initial large release of Ca2+ from the endoplasmic reticulum occurs, and is followed by a series of repeated periodic and sustained [Ca2+]i bursts. The [Ca2+]i bursts are the result of an extracellular influx of Ca2+, and they are identifiable as periods of elevated [Ca2+]i having a clear maxima at least twice the basal level, and with distinct minima between burst elevations. These [Ca2+]i bursts were found to drive greater NO production, and this process was entirely dependent on connexin 43 (Cx43) gap junction (GJ) function. Given there is no known mechanism coupling TRPC3 opening to Cx43 itself, these findings led us to question just how do changes in cell–cell coupling via Cx43 bring about enhanced TRPC3/IP3R2 coupling and that underpins sustained [Ca2+]i bursting?

One possibility, of course, is that Cx43 mediates the transfer of Ca2+ or IP3 between cells and there is no need for other explanations. However, published studies on other endothelial cell types have shown agonist‐stimulated responses that include reciprocal periodicity of both [Ca2+]i and *V*_m_ that are synchronous (Laskey et al. 1992; Dawson et al. 2006; Sheng and Braun 2007), and early studies on TRPC3 found the channels to be sensitive to membrane potential (*V*_m_) (Kiselyov et al. 1998). Given enhanced Cx43 connectivity during pregnancy, possible changes in electrical coupling may be capable of being communicated across the endothelium, and so facilitating and coordinating TRPC3 opening events. An alternative based on other studies in the broader cardiovascular field suggest endothelial Ca2+‐activated K+ channels (KCa) could conceivably respond to changes in [Ca2+]i in a manner that also influences membrane potential (*V*_m_), but the difference is this would be a *consequence* of gap junction‐enhanced Ca2+ bursting (Ledoux et al. 2008; Félétou 2009; Kochukov et al. 2014). Given both these possibilities, we set out to examine if enhanced [Ca2+]i bursting and synchronization of bursting in P‐UAEC: (1) is due to corresponding dynamic changes of *V*_m_ initiating in individual cells which can spread across the endothelium monolayer to synchronize and coordinate burst function; (2) is not directly related to dynamic changes in *V*_m_ and that any changes in *V*_m_ observed at all may be a secondary event or even serve a completely different function. To that end, we herein report our investigations of parallel changes in ATP‐stimulated Ca2+ and observed *V*_m_ responses in UAEC from nonpregnant and pregnant ewes. Specifically, we have for the first time characterized the basic electrical properties of individual P‐UAEC and NP‐UAEC using electrophysiology, which can now be used as baseline references for any future studies involving channels that may alter *V*_m_ or ion flow in these cells. Secondly, acknowledging the importance of cell–cell communication in UAEC, we have investigated responses from cells in confluent monolayers using dual‐fluorescence microscopy to observe real‐time changes of [Ca2+]i and corresponding *V*_m_ in an ATP dose‐dependent manner. The outcome of our studies suggest that while acute and sustained phases of [Ca2+]i signaling are each regulated dose‐ and time‐dependently by ATP, *V*_m_ is not strongly or dynamically coregulated in either P‐UAEC or NP‐UAEC. Our data further suggest that *V*_m_ most clearly associates with overall Ca2+ mobilization rather than the [Ca2+]i being tightly controlled by more dynamic shift in *V*_m_. Despite the observation of only minor changes in *V*_m_, we have also observed P‐UAEC are capable of mounting Ca2+ waves across cell monolayers, while these waves are far less apparent or even completely lacking in monolayers of NP‐UAEC. These findings considerably further our understanding of the mechanisms underlying endothelial cell adaptation of Ca2+ signaling that is necessary for enhanced vasodilator production in pregnancy, and may also provide mechanistic insight into how this may fail in preeclampsia and other hypertensive disorders involving endothelial vasodilatory dysfunction.

## Materials and Methods

### Materials

Minimum essential medium (MEM), Fura‐2 AM, Pluronic Acid, DIBAC4(3), and round 13‐mm glass cover slips were obtained from Thermo Fisher Scientific (Grand Island, NY or Fitchburg, WI). ATP (disodium salt), electrophysiology reagents, and all other chemicals were from Sigma‐Aldrich (St. Louis, MO) unless noted otherwise. CaCl_2_ was from Calbiochem (San Diego, CA); 35‐mm dishes with glass‐bottom coverslip windows were from MatTek Corp. (Ashland, MA). Electrophysiology pipettes were made from 1.65‐mm‐OD glass capillary tubing (Warner Instruments, Hamden, CT).

### Solutions

Kreb's solution used in imaging and electrophysiology (bath solution) studies comprised of KCl (5 mmol/L), NaCl (125 mmol/L), HEPES (25 mmol/L), glucose (6 mmol/L), CaCl_2_ (2 mmol/L), KH_2_PO_4_ (1 mmol/L), and MgSO_4_ (1 mmol/L) at pH 7.4. ATP stock solutions were freshly made at 100× concentration and diluted to final concentration upon addition.

### Isolation of UAEC

Animal handling protocols for experimental procedures were approved by the University of Wisconsin‐Madison Research Animal Care Committees of the School of Medicine and Public Health and of the College of Agriculture and Life Sciences and followed the guidelines for humane treatment and euthanasia of laboratory farm animals as recommended by the American Veterinary Medicine Association. Uterine artery endothelial cells were obtained from pregnant ewes (120–130 days gestation) and nonpregnant ewes (staged to the luteal phase) of Polypay and mixed Western breed during nonsurvival surgery as previously described (Bird et al. 2000; Yi et al. 2011) and grown in MEM containing 20% FBS, 1% penicillin‐streptomycin, and 4 *μ*g/mL gentamycin (growth medium) to passage 3 before trypsinization and storage in liquid nitrogen in the same growth medium containing 10% dimethylsulfoxide (DMSO) from Sigma‐Aldrich (St. Louis, MO). For all subsequent imaging experiments, a pool of cells from four animals each was created (NP and P pools). Passage 3 cells from four individual animals were thawed and grown in growth medium to near confluence, and then combined and split 1:35 for freezing at passage 4 in growth medium with 10% DMSO. These cell pool aliquots were then used for all subsequent cell imaging studies as required.

### Electrophysiology

Whole‐cell configuration of patch clamp electrophysiology was performed to determine the basic electrical properties of P‐UAEC and NP‐UAEC. Briefly, cells were cultured in 20% FBS‐MEM based growth medium on round glass coverslips or in glass‐bottom dishes and then mounted to the microscope recording platform. Cells were perfused with Krebs' solution at room temperature using a gravity fed multi‐line perfusion system controlled by electronic shut‐off valves at a rate of ~1 mL/min. Healthy individual, isolated cells were chosen for patch‐clamping through visual confirmation at 60× magnification. Fire polishing of pipette tips resulted in an impedance of 2–8 mol/L·Ω. Perforated patch pipette solution contained KCl (30 mmol/L), K‐gluconate (83 mmol/L), K2HEPES (10 mmol/L), K2EGTA (5.5 mmol/L), CaCl_2_ (0.5 mmol/L), and MgCl_2_ (4 mmol/L) at pH 7.2. Backfill solution included 120 *μ*g/mL amphotericin B. Standard pipette solution contained KCl (30 mmol/L), K‐gluconate (83 mmol/L), K2HEPES (10 mmol/L), K2EGTA (5.5 mmol/L), CaCl_2_ (0.5 mmol/L), MgCl_2_ (4 mmol/L), K2ATP (4 mmol/L), and Na2GTP (0.1 mmol/L) at pH 7.2. High K+ solution contained KCl (100 mmol/L), NaCl (30 mmol/L), HEPES (25 mmol/L), glucose (6 mmol/L), CaCl_2_ (2 mmol/L), KH_2_PO_4_ (1 mmol/L), and MgSO_4_ (1 mmol/L) at pH 7.4. Recordings were carried out on single, voltage‐clamped cells using an Axopatch 200B amplifier system, Digidata1440A digitizer, and pCLAMP 9.0 data acquisition software (Molecular Devices, Sunnyvale, CA). Voltage steps or ramp protocols changed the membrane potential from −150 to +50 mV over 1.5 sec, with in between holding potential of −60 mV. Additional stock solutions of 100 *μ*mol/L ATP and 100 mmol/L K+ were diluted with Kreb's just prior to perfusion. Curve fitting and statistical analysis was completed using Microsoft Excel and SigmaPlot 12 (Systat Software, San Jose, CA) with a voltage correction of −9 mV applied to all values due to the existence of a liquid junction potential between the bath solution and pipette tip causing a flowing saturated KCl bridge.

### Fluorescent dye imaging

Imaging control and image acquisition was achieved with MetaFluor 7.7.9 software (Molecular Devices, Sunnyvale, CA). Calibration of [Ca2+]i for Fura‐2 studies was achieved by establishing a standard curve using buffer solutions of known free [Ca2+] (Molecular Probes, Grand Island, NY) and Fura‐2 free acid.

Pooled cells frozen at passage 4 were thawed and grown on 35‐mm glass‐bottom dishes in standard growth medium to 90% or greater confluency. Cells were loaded in Kreb's solution (1 mL) containing 10 *μ*mol/L Fura‐2 AM + 0.05% pluronic acid and incubated at 32°C in the dark for 45 min. Cells were then rinsed twice with Kreb's solution containing 250 nmol/L DIBAC4(3) to remove excess Fura‐2 AM. After rinsing, 2 mL of 250 nmol/L DIBAC4(3) were added to the dish and cells were allowed 30 min at room temperature in the dark to complete Fura‐AM hydrolysis. At the end of loading, cells were placed on a heated microscope stage using a 35 mmol/L culture dish heater (DH‐35, Warner Instruments, Hamden, CT) and maintained at 23–26°C by a temperature controller box (TC‐324B, Warner Instruments, Hamden, CT). A specific patch of tightly grouped cells was identified under 380 nm and/or 480 nm UV excitation using a Nikon Ti‐E inverted microscope equipped with a 20× phase/fluor objective and a 175W Lambda LS xenon arc lamp (Sutter Instrument Co, Novato, CA). Individual cells (59 cells) were selected and imaged at 2‐sec intervals via excitation filters alternating 340 nm and 380 nm (300 and 150 milliseconds per excitation) for Fura‐2 responses and additionally at 6‐sec intervals via 480 nm (100 milliseconds per excitation) for DIBAC4(3). Emission light passed through a 505 nm dichroic mirror, and was filtered at 520 nm via a second filter wheel (Lambda 10‐3; Sutter). Responses were captured with a Cascade 512B high‐speed camera (Photometrics, Tucson, AZ) with data acquisition controlled by Metafluor software. Baselines were observed for 5 min prior to stimulating UAEC with ATP (0, 1, 3, 10, 30, or 100 *μ*mol/L). ATP stock was diluted to 10× in a vial using dish media, then returned to the dish, and immediately mixed to a final concentration by manual pipette suction/expulsion with stimulation responses being recorded for 30 min. Post hoc video analysis of the [Ca2+]i response was later performed on 340/380 ratio TIF image files recorded in MetaFluor (pseudocolor, brightness, and contrast 50%, display min 0, display max 2). Images were merged into a single AVI video file using MetaFluor and viewed at 30.7 fps with ImageJ 1.50i software (National Institutes of Health, USA).

### Statistics

[Ca2+]i and *V*_m_ data at either fixed time points, or as “area under the curve” (AUC), were compared using student's *t*‐test, or paired *t*‐test as appropriate. For regression analysis, where a dose response was expected, a Sigmoidal fit was used. Other datasets were analyzed using a linear or nonlinear regression based on the equation which best fit the data (all tests performed in SigmaPlot V13.0). Differences in *P*‐value <0.05 were considered significant.

## Results

### Electrophysiology

#### Baseline electrical properties of UAEC

**Figure 1:**
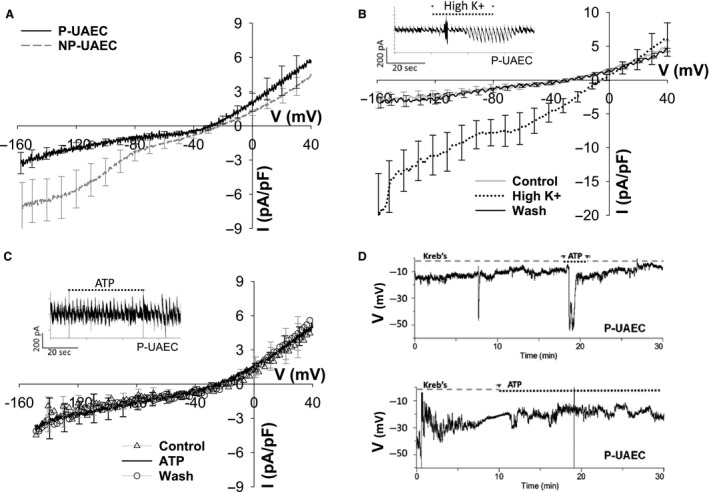
Basic electrical properties of UAEC. (A) *I*–*V* response of UAEC (*P* = 4, NP = 4) during whole‐cell voltage ramp (−160 to +40 mV 1.5 sec, holding 0.5 sec at −60 mV). Cells were perfused in Kreb's buffer bath solution, ~1 mL/min. No statistical difference between *P* versus NP current, tested at 10‐sec intervals. (B) P‐UAEC (*n* = 4) exposed to high K+ (100 mmol/L) bath solution showed a reversible strong inward rectifier current. *Inset* – continuum of individual 2‐sec ramp responses in a representative cell. (C) The averaged P‐UAEC (*n* = 3) *I*–*V* response to ATP (100 *μ*mol/L) solution was not significantly different compared to control buffer response. *Inset* – continuum of individual 2‐sec ramp responses in a representative cell. Tracings (A–C) are mean ± SEM. (D) Representative tracings from two different P‐UAEC. Cells show fluctuating *V*_m_ under whole‐cell current clamp conditions in perfused Kreb's buffer solution and ATP exposure does not consistently induce hyperpolarization in P‐UAEC.

Whole‐cell patch clamp recordings were undertaken to establish baseline biophysical parameters for isolated P‐UAEC (*n* = 37) and NP‐UAEC (*n* = 7). We determined the average membrane potential (*V*_m_) of P‐UAEC, which was −22.0 ± 2.25 mV and for NP‐UAEC, was −23.9 ± 2.20 mV with values corrected by −9 mV for liquid junction potential. The membrane capacitance (*C*_m_) values were P‐UAEC (17.1 ± 2.33 pF) and NP‐UAEC (16.1 ± 1.89 pF), and values of membrane resistance (*R*_m_) were P‐UAEC (3.96 ± 0.726 GΩ) and NP‐UAEC (2.78 ± 0.810 GΩ). None of these baseline electrical parameters were statistically different between P‐UAEC and NP‐UAEC at low cell density. Application of voltage ramp protocols (Fig. 1A) showed both P‐UAEC and NP‐UAEC display outward current at depolarizing potentials, a plateau region between −80 and −40 mV, and average current responses that showed separation at more hyperpolarized potentials, but whose slopes were not statistically different from each (*slope* −40 to −80 mV: *P* = 0.007 ± 0.004, NP = 0.035 ± 0.012, *P* = 0.072; *slope* −80 to −160 mV: *P* = 0.0305 ± 0.0112, NP = 0.0620 ± 0.0190, *P* = 0.20). Additionally, the average inward current responses between P‐UAEC and NP‐UAEC were compared at 20 mV intervals with no significant differences noted (*P* = 0.087 at −140 mV, *P* = 0.070 at −120 mV, *P* = 0.080 at −100).

#### P‐UAEC show minor fluctuations of *V*_m_

Individual cells were exposed to either an elevated K+ solution (100 mmol/L) or to ATP (100 *μ*mol/L), and we used *voltage ramp* whole‐cell methods to monitor the current response. Figure 1B shows an elevated K+ solution shifted P‐UAEC's reversal potential from a control value of −24.5 ± 4 mV to −4.5 ± 3 mV with a significant increase in the amplitude of inward current signifying the presence of inward rectifier channels in P‐UAEC. Both the current amplitude and *V*_m_ values completely reversed upon reintroduction of basal Kreb's buffer solution. Under our recording conditions, exposure to ATP (Fig. 1C) did not consistently change the current amplitude nor shift the *V*_m_ compared to the control response and the averaged ATP response was no different than the control average.

**Figure 2:**
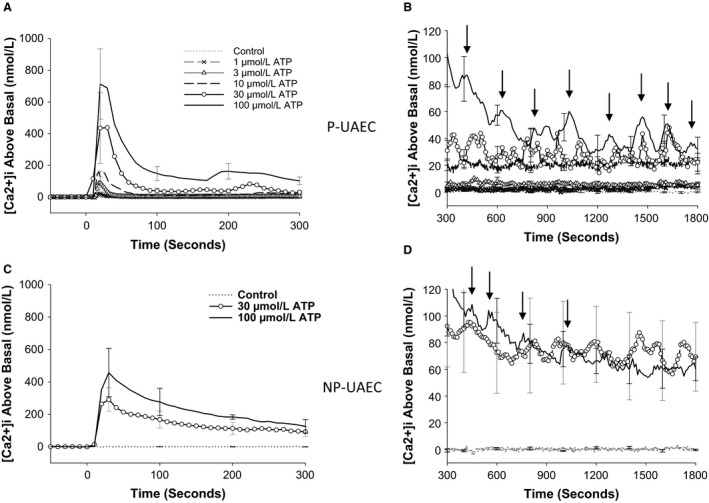
P‐UAEC and NP‐UAEC show unique [Ca2+]i responses to ATP stimulation. ATP stimulation results in a larger initial [Ca2+]i peak followed by a series of [Ca2+]i bursts during the sustained phase. The dish‐averaged tracings (B vs. D) show synchronization of P‐UAEC > NP‐UAEC as multiple cells averaged together maintain clear patterns of [Ca2+]i bursting in P‐UAEC. (A) ATP dose‐dependently increased the height and width of the initial [Ca2+]i peak in P‐UAEC. A clear second burst is noted at 200–300 sec with higher ATP doses (30 and 100 *μ*mol/L). (B) ATP dose‐dependently increased both sustained [Ca2+]i elevation and the frequency of individual [Ca2+]i bursts (gray arrows) in P‐UAEC. (C) ATP stimulation appears to also dose‐dependently increase the size of the initial [Ca2+]i peak of NP‐UAEC, but maximum elevation is less in NP than P and lacks the distinct second burst. (D) ATP dose dependency on sustained [Ca2+]i elevation or [Ca2+]i burst frequency is not readily apparent in NP‐UAEC. Averaged tracings are the mean response of *n* = 5 dish averages ± SEM (dish average = 59 cells per dish).

**Figure 3:**
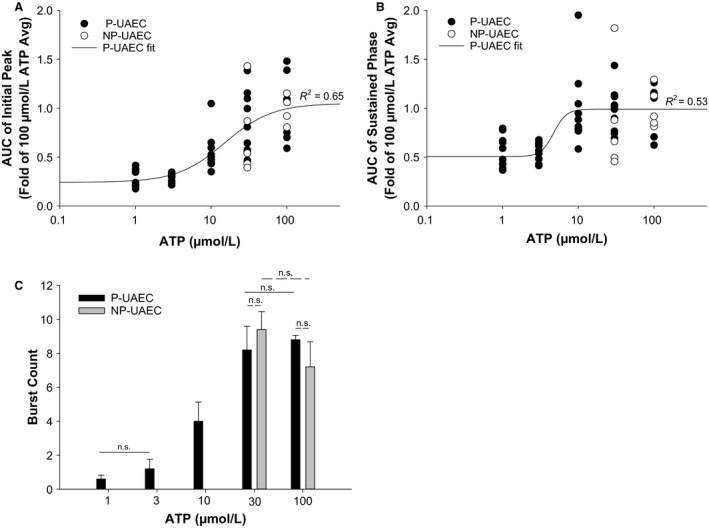
ATP Dose Response Effects on AUC of Initial and Sustained [Ca2+]i in UAEC. P‐UAEC were stimulated with ATP (1, 3, 10, 30 or 100 *μ*mol/L) and the observed [Ca2+]i elevation over time was reported as Area Under the Curve (AUC). Each dish response is reported as fold of the respective group's average 100 *μ*mol/L response (*n* = 59 cells per dish, *P* = 6–10/NP = 5 dishes per dose). (A) For the *initial* [Ca2+]i peak, the sigmoidal curve fit of the ATP versus AUC continues to increase with ATP dose as expected. (B) The *sustained* [Ca2+]i phase sigmoidal curve fit of ATP versus AUC shows maximal [Ca2+]i response occurred even at submaximal doses of ATP. All regression fits are *P* < 0.0001. (C) In P‐UAEC, ATP dose‐dependently effects the number of [Ca2+]i bursts observed in dish‐averaged tracings during the sustained phase (*n* = 5 dishes per dose ± SEM). P‐UAEC burst count data follow a more progressive dose–response than seen in “B” AUC of the sustained phase. No significant difference is noted for P‐UAEC versus NP‐UAEC dish count burst average for 30 or 100 *μ*M ATP.

P‐UAEC *V*_m_ responses were also recorded using *current clamp* whole‐cell methods in a limited number of isolated cells. We observed *V*_m_ predominantly hovered around a steady baseline in cells perfused with control media (Krebs's buffer), but hyperpolarization and depolarization events or periods of fluctuating *V*_m_ sometimes occurred (Fig. 1D: upper graph 7.5 min, bottom figure 0–3 min). Switching the media to elevated K+ solution did not cause shifts in *V*_m_ that were of greater magnitude beyond the random *V*_m_ changes observed under baseline conditions (data not shown). Additionally, exposure to ATP during perforated patch current clamp recordings did not consistently induce a change in *V*_m_ (1 of 3 cells), but when hyperpolarization resulted, the event lasted <60 sec as seen in Figure 1D (top).

### Fluorescent imaging of cell monolayers

#### ATP dose‐dependently affects both the initial [Ca2+]i peak and sustained phase [Ca2+]i entry

In Figure 2A and B, data are expressed as *combined dish average* responses (all cell data combined per dish, *n* = 5 dishes per dose). ATP‐stimulated P‐UAEC clearly show a dose‐dependent [Ca2+]i response for both the initial [Ca2+]i peak and for the secondary sustained [Ca2+]i phase. ATP dose‐dependently increased both the initial [Ca2+]i peak's height and the duration of the peak's downslope, and [Ca2+]i failed to completely return to baseline even showing a secondary “bump” of [Ca2+]i at 200 sec at the higher ATP doses (Fig. 2A). Of note, in the sustained phase response of P‐UAEC (Fig. 2B), the return from an elevated [Ca2+]i steady state is dose dependent and includes periodic [Ca2+]i elevations that are clearly pronounced above the elevated baseline at ATP dose ≥30 *μ*mol/L.

When NP‐UAEC (*n* = 5 dishes per dose) were stimulated with ATP (30 or 100 *μ*mol/L), we saw a dose‐dependent increase in the initial [Ca2+]i peak (Fig. 2C), but in comparison to P‐UAEC, the initial peak was less pronounced in height with a sustained phase downslope that was more drawn out in time. While the effects of 30 *μ*mol/L versus 100 *μ*mol/L ATP were observably different for about 10 min, thereafter the continued elevation of the sustained [Ca2+]i phase became less distinct (Fig. 2D).

In Figure 3, we further analyzed ATP dose dependency in P‐UAEC by calculating area under the curve of [Ca2+]i for both the initial peak and sustained phase ATP‐stimulated responses. Dish AUC averages (*n* = 6–10 dishes per dose) were expressed as a fold of the averaged 100 *μ*mol/L ATP AUC response and data were fit using a sigmoidal curve. A clear ATP dose response is seen for the initial [Ca2+]i peak (Fig. 3A) with a calculated EC50 of 15.1 ± 5.94 *μ*mol/L and a Hill slope value of 1.4 ± 0.61. The threshold dose and maximal effective dose of ATP for the initial peak are entirely consistent with our previous publication (Di et al. 2001; Gifford et al. 2003). The sustained phase response (Fig. 3B) showed a smaller EC50 value (4.7 ± 3.5 *μ*mol/L) and had a very abrupt increase between 1 and 10 *μ*mol/L ATP before the response plateaued. By this analysis at least, sustained phase Ca2+ seemed to occur as an “all or nothing” event triggered within a very narrow range of ATP stimulation. Nonetheless, when we examine dose dependency of synchronous bursting (Fig. 3C) as observable bursts in dish responses that make up the averaged tracings of Figure 2B, we see a more progressive dose‐dependent increase in *synchronous* burst number count. Comparison of NP‐UAEC data from 30 and 100 *μ*mol/L ATP stimulations showed similar magnitudes of *overall* [Ca2+]i response compared to P‐UAEC for both initial peak and sustained phase AUC (Fig. 3A and B).

**Figure 4:**
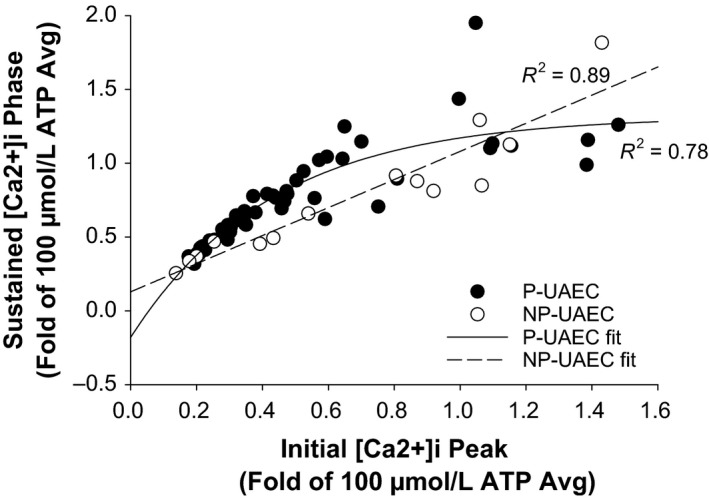
Correlation of initial [Ca2+]i peak to sustained [Ca2+]i phase. P‐UAEC were stimulated with ATP (1, 3, 10, 30, or 100 *μ*mol/L) and the average total [Ca2+]i response during initial (0–300 sec) or sustained (300–1800 sec) phase was calculated for each dish as a fold of the averaged 100 *μ*mol/L response. NP‐UAEC data from ATP stimulation (0, 30, 100 *μ*mol/L) were calculated using the respective NP‐averaged 100 *μ*mol/L response. P‐UAEC data were most accurately fitted using a nonlinear, three‐parameter, regression, while NP‐UAEC data clearly followed a linear regression. Regression fits *P* < 0.0001. P‐UAEC: 6–10 dishes per dose, NP‐UAEC: 5 dishes per dose; 59 cells per dish.

Figure 4 shows a P‐ versus NP‐UAEC difference in the relationship between the initial [Ca2+]i peak and the sustained [Ca2+]i phase. In P‐UAEC, the AUC of the initial [Ca2+]i peak appears to be causally linked to AUC of the sustained phase under minimally stimulatory conditions and a near‐linear relationship exists up to 0.5‐fold response, but with larger initial peak responses (greater than 0.5‐fold), the relationship curves to a near steady state.

#### P‐UAEC show intercellular waves of [Ca2+]i

**Figure 5:**
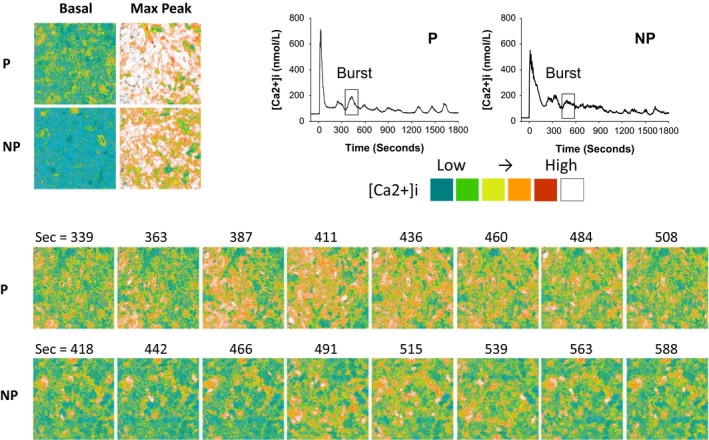
The appearance of synchronous [Ca2+]i bursts in P‐UAEC in a field of cells. UAEC stimulated with ATP (100 *μ*mol/L) show [Ca2+]i bursting within individual cells during the sustained phase and the responses are more synchronous/coordinated between neighboring cells in P‐UAEC than in NP‐UAEC. The ATP‐stimulated Ca2+ response of a single sustained phase [Ca2+]i burst (boxed region in tracings) is shown as a series of snapshots acquired during a 35‐min video recording. Top left: P‐UAEC and NP‐UAEC images show nearly all cells initially responded to ATP. The basal [Ca2+]i prior to ATP stimulation is compared to the corresponding maximum [Ca2+]i response at the height of the initial [Ca2+]i peak. Bottom series: P‐UAEC image at time = 363 sec shows a heightened [Ca2+]i response from multiple cells in the SW corner of the image, followed by a synchronized response between multiple cells that appeared as a wave of increased [Ca2+]i moving in a NE direction across the field of cells as time progressed. NP‐UAEC images show increased [Ca2+]i occurs in an asynchronous manner. Elevation begins in several more discrete points at time = 442, and shows little evidence of a coordinated or directional propagation across the field of cells. See supplementary files for corresponding video clips of the P‐UAEC (S1) and NP‐UAEC (S2) responses.

Given our previous observations that gap junction communication is greater in P‐UAEC and underpins more synchronous behavior (Yi et al. 2011), we undertook whole field video image analysis (Fig. 5) to observe both the extent to which cells respond to 100 *μ*mol/L ATP stimulation and how coordinated their responses were with each other as a monolayer. At the height of the initial [Ca2+]i peak (Fig. 5, top left) the P‐UAEC preparation shows a far more continuous array of cells with a maximal [Ca2+]i response compared to the NP‐UAEC preparation's response. The series of video snapshots (Fig. 5, bottom) demonstrate how [Ca2+]i elevation changes across the field of P or NP cells during a sustained phase [Ca2+]i burst lasting nearly 3 min. In P‐UAEC, the coordinated [Ca2+]i increase is identified as initiating at the SW corner of the observation frame (time = 363) and the synchronized response progresses as a wave of elevated [Ca2+]i moving across the entire field of cells in a NE direction. While individual cells still show sporadic periods of elevated [Ca2+]i, there is a clear coordinated wave of [Ca2+]i that moves across the field of P‐UAEC. Captured video images from NP‐UAEC show that the [Ca2+]i burst is a composite of broadly scattered groups of cells responding in an asynchronous manner, with NP‐UAEC showing several discrete origination points of [Ca2+]i elevation but little evidence of coordinated wave propogation across the field of cells. [See Video S1 and S2 to view full video files showing “wave” vs. asynchronous Ca2+ mobilization in UAEC].

#### Basal versus ATP‐stimulated *V*_m_ change in P‐UAEC

**Figure 6:**
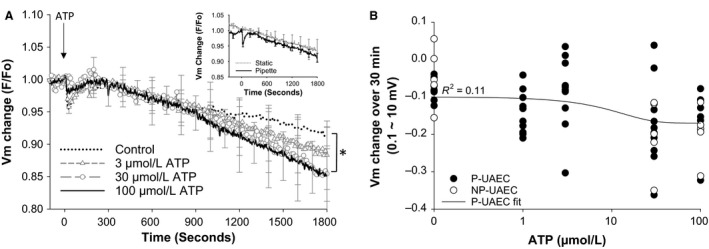
P‐UAEC stimulation by ATP results in *V*_m_ hyperpolarization. P‐UAEC were monitored for *V*_m_ change in response to ATP stimulation (0, 3, 30, or 100 *μ*mol/L ATP) using the potentiometric dye DIBAC4(Dawson et al. 2006). (A) Change in *V*_m_ ‐associated fluorescence was seen upon ATP addition. Simulation of cells by 30 or 100 *μ*mol/L ATP significantly hyperpolarized the response over 30 min compared to control (0 *μ*mol/L ATP) *P < 0.05. *Inset –* shows the change in *F*/*F*_o_ due to photobleaching for cells observed under static basal control media conditions versus the response when basal control media was added by pipette addition. (B) The average net *V*_m_ change over 30 min was evaluated for each dish of UAEC. P‐UAEC data were fit using a sigmoidal three‐parameter curve. Even with a best regression fit, the correlation between *V*_m_ change and [ATP] is weak (*P* = 0.089). NP‐UAEC data are shown for reference. Tracings and data points are the mean dish response ± SEM;* n* = 9–10 dishes, 59 cells per dish.

In establishing baseline *V*_m_ values in isolated UAEC via electrophysiology, it became apparent that manual analysis of individual cells was not sufficient to address our question of possible changes in *V*_m_ on ATP stimulation and their relationship with [Ca2+]i. We therefore switched our approach to a more efficient method, and simultaneously analyzed *groups* of endothelial cells in a confluent state using the voltage‐sensitive dye DIBAC4(3). DIBAC4(3) is capable of reporting membrane potential change in nonexcitable cells, and studies show that a 1% change in DIBAC4(3) fluorescence approximates a 1 mV change in membrane potential (Brauner et al. 1984; Epps et al. 1994; Klapperstuck et al. 2009). P‐UAEC showed ATP dose dependency of [Ca2+]i in Figures 2 and 3, but corresponding changes in *V*_m_ ‐related fluorescence measured in the same cells showed little difference for the ATP concentrations tested (Fig. 6A). It was noted in preliminary studies that use of DIBAC4(3)‐loaded UAEC (inset Fig. 6A) was not easy as they underwent a linear decrease in apparent *V*_m_ fluorescence due to photobleaching, and physically pipetting the Kreb's buffer control media caused an observable dip in *V*_m_ ‐related fluorescence (unrelated to ATP) which recovered to baseline by 5 min. The majority of individual P‐UAEC showed decreasing DIBAC4(3) fluorescence over 30 min (data not shown), and the overall dish‐averaged *V*_m_ response trended in the hyperpolarized direction. On agonist stimulation, UAEC did not show added oscillatory *V*_m_ behavior as has been reported in some types of endothelial cells (Laskey et al. 1992). A curve fit analysis of [ATP] against net *V*_m_ change over 30 min (Fig. 6B) suggested only a small agonist‐specific enhancement of hyperpolarization moving from 0 to 30 *μ*mol/L ATP, which became significantly different from control at ≥30 *μ*mol/L ATP (0* μ*mol/L −8.0 ± 1.0 mV, 30 *μ*mol/L −0.17 ± 3.6 mV, 100 *μ*mol/L −16 ± 3.5 mV; *P* < 0.05). Overall, a “dose dependency” curve fit for this small response failed to achieve significance. NP‐UAEC *V*_m_ data are also plotted on the curve in Figure 6B, and shows similar results as P‐UAEC with 30 and 100 *μ*mol/L ATP responses significantly different from control. The 100 *μ*mol/L ATP‐stimulated net *V*_m_ change in P‐UAEC (−16 ± 3.5 mV) was not statistically different from NP‐UAEC (−20 ± 3.2 mV) at 30 min. Indeed, UAEC appear to tightly maintain *V*_m_ even under artificially challenged situations, and ATP only slightly changes *V*_m_ at the very highest dose of ATP.

#### Relationship between ATP‐stimulated [Ca2+]i and *V*_m_ change

**Figure 7:**
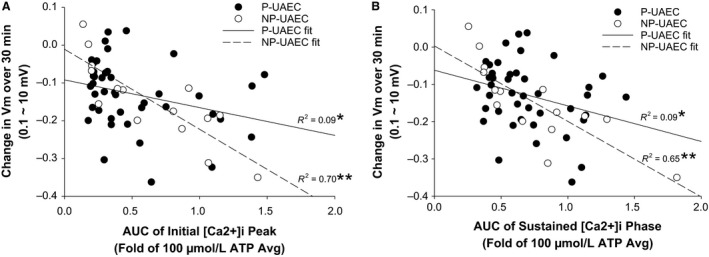
Correlation of area under the curve (AUC) of [Ca2+]i to changes in *V*_m_. P‐UAEC stimulated with ATP (0, 1, 3, 30, or 100 *μ*mol/L) or NP‐UAEC (0, 30, 100 *μ*mol/L) show an increase in total [Ca2+]i during both an initial (0–300 sec) and sustained (300–1800 sec) phase. The AUC was calculated for each dish as a fold of the respective P or NP‐averaged 100 *μ*mol/L response and was plotted against the corresponding change in *V*_m_ over 30 min. P‐UAEC and NP‐UAEC data were fit using a linear curve. (A) The relationship between net *V*_m_ change and the AUC of the *initial* [Ca2+]i peak is stronger in NP‐UAEC than in P‐UAEC. Small changes in AUC in P‐UAEC were associated with a span of minimal to maximal *V*_m_ changes for individual dishes. (B) Data points are widely scattered with little correlation between the AUC of the *sustained* [Ca2+]i phase and *V*_m_ change in P‐UAEC, but NP‐UAEC show a stronger correlation between elevated [Ca2+]i and *V*_m_ change. Data points are dish averages (*n* = 9–10 dishes per dose, 59 cells per dish). Regression fits are significant at *P < 0.05 for P‐UAEC and **P < 0.0005 for NP‐UAEC.

Given that ATP could dose‐dependently alter [Ca2+]i, and only to a lesser extent magnify hyperpolarization in P‐UAEC, we next asked if [Ca2+]i was directly correlated with/could actually be directly responsible for the small *V*_m_ change. A linear curve fit analysis of AUC of [Ca2+]i versus net *V*_m_ change in P‐UAEC did achieve a significant correlation (*P* < 0.05). Nonetheless, Figure 7A shows even a small increase in the initial [Ca2+]i peak (<25% of max) resulted in a variable range of *V*_m_ values that spanned from depolarized to hyperpolarized shifts in *V*_m_. Likewise, the significant correlation of AUC of the sustained [Ca2+]i phase to net *V*_m_ change (Fig. 7B) also still resulted from a wide spread of responses. Nonetheless, the greatest shifts of net *V*_m_ change were not seen unless AUC of [Ca2+]i reached levels 50% or greater of the averaged maximum response. In comparison, NP‐UAEC linear correlations between AUC and *V*_m_ change were more highly significant (*P* < 0.0005) and more predictive in showing greater hyperpolarization as AUC increased for both the initial peak and sustained [Ca2+]i phases.

## Discussion

Our initial electrophysiology characterization of isolated UAEC showed classic endothelial electrical behavioral properties. Specifically, isolated UAEC were seen to have *V*_m_ values that fell within the expected range of *V*_m_ values (−52 mV to −16 mV) reported in other endothelial isolated cell studies (Vargas et al. 1994; Yang et al. 2003; Ledoux et al. 2008). One limitation of our electrophysiology study was we were unable to accurately determine *V*_m_ values of UAEC in a confluent monolayer, as their flat morphology at high density made them very difficult to consistently patch clamp and this was further confounded by voltage clamp errors due to contact between neighboring cells. Despite this limitation, other endothelial cells such as human umbilical vein endothelial cells (HUVEC) which have a more cobblestone morphology have been reported to show slightly more negative *V*_m_ values at high density compared to low cell density (−24 mV vs. −16 mV) (Vargas et al. 1994) and it is likely that UAEC would also have slightly more negative resting *V*_m_ values in a confluent state. Nonetheless, our electrophysiology data are important to the understanding of mechanistic function in UAEC and similar current responses for P‐UAEC and NP‐UAEC imply membrane channels sensitive to voltage regulation should have comparable activation capabilities in both the nonpregnant and normal healthy pregnant state. This is not surprising as both P‐UAEC and NP‐UAEC originate from the same region of the uterine vasculature, follow similar growth patterns during cell culture, and generally shown no differences in expression levels of key signaling proteins (Gifford et al. 2006a,b).

Despite electrical similarities, NP‐UAEC showed greater variance between cells which deviated from the P‐UAEC average response at more hyperpolarized potentials. These findings suggest that an unidentified pregnancy‐specific mechanistic shift is present which lends toward synchronization of cell behavior in P‐UAEC. Regulated current control in P‐UAEC was also apparent in ATP experiments in that exposure did not change the nature of the *I*–*V* curve, and current clamp protocols revealed few occurrences of *V*_m_ change by ATP. We did also see differences between P‐UAEC and NP‐UAEC responses for *V*_m_ and [Ca2+]i when observed in a confluent state using fluorescent dyes. This fits the paradigm of more recent studies, which suggest that it is the active open state of gap junctions formed by Cx43 of *adjoining* UAEC that principally determines Ca2+ signaling underlying pregnancy‐enhanced vasodilatory function (Yi et al. 2010; Morschauser et al. 2014).

Our initial question going into the high‐density dual‐imaging studies was whether ATP promoted a dose‐dependent change in a *V*_m_‐dependent signal. We also hypothesized that *V*_m_ change was tightly temporally correlated with [Ca2+]i bursting as a means for directly enhancing Ca2+ influx through TRPC3 channels in UAEC. Thus, our expectation was that *V*_m_ responses would generally follow an inverse oscillatory trend with [Ca2+]i. UAEC showed an immediate [Ca2+]i response upon ATP stimulation, yet the *V*_m_ response did not coincide in timing or form with [Ca2+]i bursting events, as anticipated. We therefore conclude that the initial substantial rise in [Ca2+]i that occurs upon ATP stimulation solely reflects the Ins(1,4,5)P3‐mediated release of Ca2+ from the ER as proposed previously (Gifford et al. 2006a,b), and that any corresponding changes in ATP‐stimulated [Ca2+]i bursting that follow the initial peak phase cannot be the direct result of a global dynamic change in *V*_m_. Such observations are consistent with our prior report that L‐type Ca2+ channel proteins are present in these cells, yet play no role in capacitative entry (Gifford et al. 2003). Even if functional L‐type Ca2+ channels are present in the plasma membrane, the cell fails to undergo a change in *V*_m_ sufficient to activate them.

The small and more gradual/progressive changes in *V*_m_ we did observe appeared to more closely relate to total mobilization of [Ca2+]i rather than the ATP dose applied to UAEC. This suggests that agonist‐dependent changes in *V*_m_ are not so much under the direct control of P2Y2 receptor occupation in UAEC, and are instead under the control of other channels acting downstream of/subsequent to the Ca2+ signaling response. Certainly, K+ channels could produce the weaker *V*_m_ changes we observed, and channels such as KCa have been shown to be active in at least some endothelial cells (Sheng and Braun 2007; Ledoux et al. 2008; Kohler and Ruth 2010). Additionally, a strong inward rectifier current was identified in P‐UAEC suggesting that cells responding to K+ efflux from underlying arterial smooth muscle cells during pregnancy could influence K+ channel function. It is appropriate now to ask if altered K+ channel activity may be part of the enhanced ability of P‐UAEC to generate synchronous and more sustained [Ca2+]i bursting in pregnancy. Such knowledge is relevant to our understanding of diseased states of pregnancy such as preeclampsia, where we have previously shown that preeclamptic‐derived HUVEC responses of [Ca2+]i bursting and NO production are similarly reduced well below levels observed in normal healthy pregnant states (Krupp et al. 2013).

Given our failure to detect any substantial dynamic *V*_m_ oscillation otherwise reported in other endothelial cells, we must also ask if DIBAC4(3) is simply unreliable as a monitor of *V*_m_ change in UAEC, or if progressive *V*_m_ hyperpolarization is indeed a property of UAEC that differs from observations in HUVEC or other endothelial cells. We found DIBAC4(3) responses are subject to mild photobleaching, so for prolonged recordings such as ours, DIBAC4(3) may not *optimally* report *V*_m_ change. Pipette media addition itself also resulted in a specific transient fluorescence response artifact. Nonetheless, both of these artifacts in the absence of ATP were constant across observations, and could be identified and corrected for. The observed *V*_m_ changes seen with added *agonist* were over and above these artifacts, and at 30 *μ*mol/L and 100 *μ*mol/L ATP became significantly greater than no agonist control responses. This leads us to conclude that the ATP‐stimulated *V*_m_ responses are small but real events in UAEC. Unlike the clear dose‐dependent changes in [Ca2+]i in the exact same cells, an analysis of ATP dose dependency on *V*_m_ failed to achieve a significant overall curve fit. Given such a weak relationship between ATP dose and change in *V*_m_, the question then follows if *V*_m_ changes are not significantly related to receptor occupancy (i.e., agonist dose applied), then are they secondary to changes in [Ca2+]i? Regression analyses of the observed *V*_m_ change versus total AUC of [Ca2+]i shows clearly there is a significant correlation between the two variables for P‐UAEC, and even more so for NP‐UAEC. This stronger correlation again is the clearest evidence that it is changes in [Ca2+]i that drive *V*_m_ directly in UAEC, and this alters with pregnancy. Given the whole‐cell nature of our initial observations, and the limitations of DIBAC4(3), we cannot rule out the additional possibility that greater magnitude *V*_m_ changes could be occurring within *subcellular* regions, perhaps related to local gap junction function and/or the frequency of local [Ca2+]i bursting activity within a cell. Higher magnification and higher speed imaging studies beyond this study would be necessary to determine this absolutely.

The combination of analyses in this study shows that the enhanced nature of the sustained [Ca2+]i phase response is not just *quantitative*, but also *qualitative* in nature to coordinate individual burst responses in ways beyond a simple increase in [Ca2+]i. Of note, our averaged [Ca2+]i tracings (which are a composite of multiple dishes of cells) still clearly reflect defined periods of [Ca2+]i bursting. Such periodic [Ca2+]i elevations are not only consistent in timing across multiple cells, but average number of “bumps” per monolayer reported here coincide with the number of synchronous Ca2+ bursts previously reported in individual P‐UAEC by Yi et al. (2010). The fact the pattern of averaged bumps is reproducible between dishes and visible across combined dish average data suggests that the periodic frequency is an inherent property of/due to a pacemaker within the cell *population*. In contrast, the initial peak and the sustained Ca2+ phases are less clearly delineated in NP‐UAEC and show far greater data variance when cells and dishes are averaged together. This is not only consistent with a relative lack of *synchronous* burst response in NP‐UAEC, it also suggests that any periodicity in NP‐UAEC is not inherent to the cell population or retained across dishes. There also appears to be a heightened sensitivity in P‐UAEC, which allows a submaximal stimulus to induce greater Ca2+ influx during the bursting phase, and this most likely is the consequence of the greater gap junction coupling in P‐UAEC compared to the lesser NP‐UAEC coupling level. This is reflected in the ATP dose response curves showing a gradual change across doses for the initial peak response, but an abrupt jump at a lower stimulatory dose for both AUC of [Ca2+]i and synchronized burst count in P‐UAEC. This also allows a more synchronous burst response within sheets of cells in P‐UAEC [reported in part by Yi et al. (2010, 2011) and inferred here in Fig. 2] so steepening the dose curve in a manner not seen in NP‐UAEC. Consistent with this, we also found that despite similar raw burst counts at maximal ATP doses for P‐UAEC and NP‐UAEC, video imaging shows quite clearly that P‐UAEC can respond more readily as a *synchronous* group of cells to coordinate directional Ca2+ wave propagation. In contrast, NP‐UAEC respond in a more individualistic/idiosyncratic manner, with more numerous origination points of [Ca2+]i elevation within the monolayer but overall no clear [Ca2+]i wave propagation (Fig. 5) despite similar total [Ca2+]i mobilization. Of relevance to future approaches in therapy when pregnancy‐adapted signaling fails, our data here suggest that adapted signaling in P‐UAEC is not simply a matter of increasing [Ca2+]i bursting in individual endothelial cells, it is the *nature* of endothelial Ca2+ burst signaling (periodicity, synchronization, wave propagation) that is key. Altered endothelial function in pregnancy extends to adapted *monolayer* function as cells shift to support communication beyond their own membrane boundaries and work together in harmony.

In summary, while our initial proposed hypothesis that P‐UAEC enhance and better coordinate function via dynamic changes in *V*_m_ was not supported, there are still three main findings from this study of importance: (1) the basic electrical properties of both P‐UAEC and NP‐UAEC have been described for the first time. (2) imaging studies have determined while global *V*_m_ changes that dynamically follow [Ca2+]i bursting are not observed, overall mobilization of Ca2+ may be responsible for the small secondary changes in *V*_m_ that are observed (particularly in NP‐UAEC). (3) the most significant finding, while ATP stimulation can mobilize similar amounts of Ca2+ in the sustained phase in NP‐ and P‐UAEC, the *qualitative* nature of Ca2+ signaling in P‐UAEC is what differs between NP‐UAEC and P‐UAEC responses and specifically allows [Ca2+]i wave propagation in P‐UAEC. Given our finding that it is net Ca2+ mobilization that drives secondary changes in *V*_m_ rather than the reverse, further studies are now needed to understand what subtypes of KCa channels play a role in UAEC [Ca2+]i signaling and so vasodilator production, and how this may change in pregnancy. These studies are currently underway in isolated UAEC and in intact uterine artery vessels.

## Conflict of Interest

The authors have no disclosures related to this study.

## Data Accessibility

## Supporting information




**Videos S1 and S2**. Observation of a [Ca2+]i wave propagation in P‐UAEC versus an asynchronous [Ca2+]i response in NP‐UAEC. Video files from which the snapshots shown in Figure 5 were extracted, were produced using ImageJ at a playback rate of 30.7 fps. (S1) P‐UAEC: video time 329–518 sec. The wave of elevated [Ca2+]i can be seen migrating across the field in a NE direction. (S2) NP‐UAEC: video time 408–598 sec. Individual cells show sporadic elevations of [Ca2+]i that are otherwise lacking coordination as a wave event.
